# Synthetic Biology Tools to Engineer Microbial Communities for Biotechnology

**DOI:** 10.1016/j.tibtech.2018.11.002

**Published:** 2019-02

**Authors:** Nicholas S. McCarty, Rodrigo Ledesma-Amaro

**Affiliations:** 1 Imperial College Centre for Synthetic Biology, Imperial College London, London SW7 2AZ, UK; 2 Department of Bioengineering, Imperial College London, London SW7 2AZ, UK

**Keywords:** microbial consortia, synthetic biology, bioproduction, biomaterials, synthetic microbial communities, synthetic consortia

## Abstract

Microbial consortia have been used in biotechnology processes, including fermentation, waste treatment, and agriculture, for millennia. Today, synthetic biologists are increasingly engineering microbial consortia for diverse applications, including the bioproduction of medicines, biofuels, and biomaterials from inexpensive carbon sources. An improved understanding of natural microbial ecosystems, and the development of new tools to construct synthetic consortia and program their behaviors, will vastly expand the functions that can be performed by communities of interacting microorganisms. Here, we review recent advancements in synthetic biology tools and approaches to engineer synthetic microbial consortia, discuss ongoing and emerging efforts to apply consortia for various biotechnological applications, and suggest future applications.

## Highlights

Microbial consortia exhibit advantages over monocultures, including division of labor, spatial organization, and robustness to perturbations.

Synthetic biology tools are used to construct and control consortia by manipulating communication networks, regulating gene expression via exogenous inputs, and engineering syntrophic interactions.

Synthetic biology approaches to control the behaviors of individual species within a consortium include population control, distribution of tasks, and spatial organization.

Constructing microbial consortia is enhanced by computational models, which can predict preferred metabolic cross-feeding networks and infer population dynamics over time.

Microbial biotechnology benefits from consortia due to the unique catalytic activities of each member, their ability to use complex substrates, compartmentalization of pathways, and distribution of molecular burden.

## Microbial Communities: An Emerging Paradigm in Synthetic Biology

**Microbial consortia** (see Glossary) or communities are ubiquitous in nature and useful in many areas of the bioeconomy [1]. **Natural consortia** are important in the production of foods, the recycling of micronutrients, and in maintaining the health of humans, animals, and plants [2]. Such microbial communities consist of member organisms that, together, are more robust to environmental challenges, exhibit reduced metabolic **burden** due to a division of labor (DOL) and exchange of resources, possess expanded metabolic capabilities relative to **monocultures**, and can communicate (chemically or physically) between species 3, 4, 5.

These unique features of microbial consortia make them an attractive platform for synthetic biologists that aim to modify microorganisms for biotechnological applications. Therefore, the field of synthetic biology is increasingly expanding from a focus on the engineering of single organisms to a discipline that, in addition, aims to engineer multiple species within dynamic communities. This comes, in part, due to the limitations inherent on engineering a single cellular **chassis**. For example, the incorporation of large or complex heterologous pathways in a single strain is limited by the ability to transfer long amounts of DNA efficiently into the selected microorganism, the requirement of a high level of pathway characterization, the presence of precursors and cofactors in the host cell, and the metabolic burden caused by the expression of the heterologous enzymatic machinery 6, 7. Moreover, monocultures are often more sensitive to system perturbations, such as environmental changes or contaminations, which necessitates highly controlled culture conditions and sterilization protocols 8, 9.

Such limitations can be surmounted by the construction of **synthetic microbial communities**, here defined as those composed of two or more genetically engineered cell populations [10]. This transition from engineering static monocultures to dynamic consortia presents unique challenges, but ultimately relies upon existing synthetic biology tools and approaches 11, 12.

In this review, we briefly discuss the tools used by synthetic biologists to interconnect microorganisms, highlight the dynamic behaviors that these foundational tools enable, and emphasize how relatively simple methods in synthetic biology can be used to engineer complex communities. Finally, we highlight some recent examples of biotechnology applications that are enhanced by synthetic microbial consortia.

## Synthetic Biology Tools to Engineer and Control Microbial Communities

Synthetic biologists use engineering approaches, including computational models and **modular** DNA ‘parts’, to rationally engineer living organisms [13]. Advancements in genetic engineering (including CRISPR/Cas systems for efficient gene deletions, insertions, and transcriptional control [14]) and rapid methods to assemble DNA fragments [15] enables modular components to be interconnected to build metabolic pathways and construct biological circuits to control cellular behavior [16].

**Figure 1:**
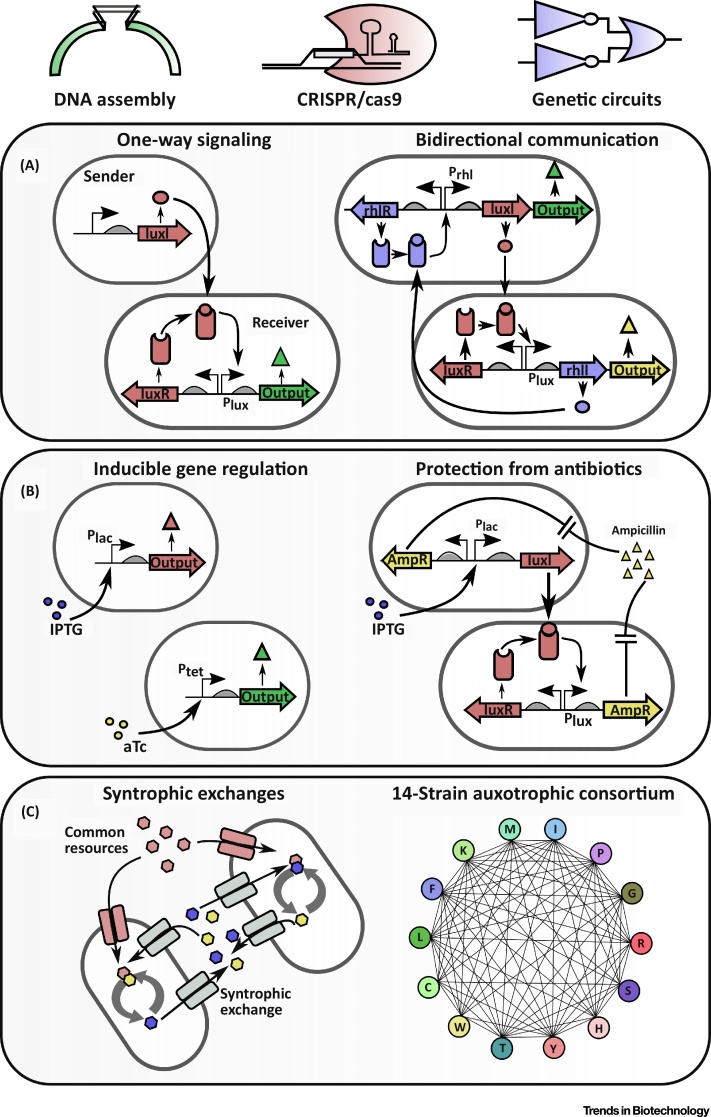
Tools to Construct Synthetic Microbial Consortia. Advancements in DNA and circuit-level assembly, CRISPR/Cas9, and other tools, enables rapid and efficient engineering of microorganisms. (A) Quorum sensing (QS) systems can be used to coordinate signaling between organisms. In the simplest case, a signaling molecule is produced from the *luxI* gene by a ‘sender’ cell, diffuses through the cell membrane, binds to its corresponding receptor (luxR), and activates transcription of the P_lux_ promoter in the ‘receiver’ cell. In bidirectional communication, each cell is both a sender and a receiver and a second, preferably orthogonal, QS system is used. Here, expression of *rhlI* produces a different signaling molecule, which binds to the corresponding receptor (rhlR) and regulates transcription of the P_rhl_ promoter. Bidirectional communication can be used to mutually activate the expression of an output in each strain. This could be antibiotic resistance genes or fluorescent reporters. Unannotated promoters are constitutively expressed. (B) Gene expression in strains within a synthetic consortium can be independently regulated via exogenous addition of inducer molecules. Isopropyl β-D-1-thiogalactopyranoside (IPTG) induces expression of the P_lac_ promoter, while anhydrotetracycline (aTc) induces expression of the P_tet_ promoter. Inducers in this example enable orthogonal control of gene expression to independently regulate the production of an output from each strain. Induction of a QS system via IPTG in one strain can also be used to trigger protective mechanisms, such as expression of an ampicillin resistance gene (AmpR), in a second strain expressing the corresponding QS receptor. This enables mutual survival of a consortium in the presence of an antibiotic, such as ampicillin. Unannotated promoters are constitutively expressed. (C) Organisms in a microbial consortium can be engineered to engage in syntrophic exchanges, in which the resources produced by one organism are used by the other and vice versa. Additionally, synthetic consortia can be assembled by deleting essential genes, typically amino acid biosynthesis genes, in each member. Survival of each member thus becomes dependent upon resource sharing from other strains in the culture. This approach enabled a 14-member *Escherichia coli* consortium to be assembled [35]. Each letter indicates an amino acid that has been deleted in the organism and straight lines indicate resource sharing between the strains.

Some synthetic biology tools, enabled by genetic engineering and DNA assembly methods, are specifically useful for controlling organisms within microbial consortia. These tools include intercellular signaling to construct communication systems between organisms, exogenous molecules to control specific population behaviors, and syntrophic interactions to build codependent networks of microorganisms (Figure 1). When utilized together, these tools facilitate the assembly and control of interactive microbial consortia.

### Intercellular Signaling to Coordinate Communication between Organisms

Synthetic microbial consortia can be constructed by adapting existing biological communication systems, such as chemical or physical communication networks, adhesion molecules, ion channels, and electricity 17, 18, 19. Quorum sensing (QS) is one such biological communication system, in which cells produce **autoinducer** molecules that act as a proxy for cell density in prokaryotic species 20, 21. As a cell population grows, cells sense the increased autoinducer concentration and regulate gene expression to control population-level behaviors.

Despite being the most common method to engineer synthetic consortia, QS systems are limited in their use as communication networks because of crosstalk at the promoter and signal levels [22]. Gram-negative organisms use *N*-acyl-L-homoserine lactones (HSL) as communication chemicals, while gram-positive organisms use autoinducing peptides. The lux and las systems, derived from *Vibrio fischeri* and *Pseudomonas aeruginosa*, respectively, are both HSL-based and are commonly used in synthetic biology due to their compatibility with *E. coli*, a model gram-negative organism. One organism (termed the sender strain) is engineered to synthesize a HSL molecule, which diffuses across cell membranes and activates gene expression in an organism (called the receiver strain) that has been engineered to express the corresponding receptor/responsive promoter pair (Figure 1A) [21]. QS-based systems can also be linked to the expression of antibiotic resistance genes or toxins, either of which can be used to modify communication between organisms in a consortium.

The **orthogonality** of QS systems has been expanded in recent years through the addition of two new QS systems for *E. coli*, *rpa* and *tra*, which have negligible signal or promoter crosstalk with one another [22], and the characterization of a six-part library of HSL-receiver devices, some of which operate orthogonally [23]. An autoinducing peptide communication system based on the *agr* QS system from *Staphylococcus aureus* also enables communication between gram-positive and -negative organisms [24].

Communication systems in higher organisms, such as yeast, can be similarly engineered by linking the production of a signaling molecule with corresponding receptors and promoters. An engineered yeast ‘sender cell’ secretes isopentenyladenine (IP), which is biosynthesized using a single enzyme derived from *Arabidopsis thaliana* and freely diffuses through cell membranes. IP binds to a cytokinin receptor in a target yeast ‘receiver cell’ and initiates a signaling cascade that results in transcription of genes from a corresponding synthetic promoter [25]. Similarly, communication systems between prokaryotes and mammalian cells can be engineered with gas-diffusible signaling molecules. *E. coli* expressing volatile acetaldehyde can activate gene expression in CHO cells with genetically encoded acetaldehyde-responsive promoters. This intercellular signaling system enables communication between organisms from disparate phylogenetic domains via gas-phase diffusion of the acetaldehyde, even when the organisms are cultured in spatially isolated wells [26]. These signaling systems can be harnessed for biotechnological applications that require communication between non-prokaryotic species.

### Exogenous Inputs to Control Cellular Behaviors

Organisms not only communicate with one another, but also sense their environment to coordinate behavior. Consequently, tools to engineer living organisms need not confine themselves to genomic modifications; synthetic biologists can also tune environmental conditions or add exogenous molecules to control gene expression and cell populations (Box 1).

Box 1 **Cellular Social Interactions in Natural and Synthetic Consortia**

Nearly all microbes live in complex communities, where the success of each organism is intimately tied to its interactions with neighbors [89]. Ecosystems of microorganisms exhibit enhanced properties relative to monocultures, such as resilience to invaders, increased robustness to environmental perturbations, and metabolic specialization, which may enhance efficiencies and growth rates of cooperating species in a microbial consortium, provided that a comparative advantage exists with respect to resources 39, 90. Community-like behavior has even been observed in isogenic populations, as in acetate production from *B. subtilis*, as bacteria specialize and occupy distinct metabolic niches [91].

Many theories have been offered to explain the increased resilience of communities relative to monocultures, including the ‘sampling hypothesis’, which suggests that the more diverse a community, the more likely it is to include an organism with heightened resistance to changing conditions, thus filling gaps left by intolerant organisms [92]. Cellular social interactions specify these ecosystem dynamics, including species coexistence and how members respond to environmental perturbations [90].

Social interactions, which include competition, predation, commensalism, **amensalism**, cooperation, and **neutralism**, are also useful in biotechnology applications of synthetic consortia [89]. Cooperative behaviors can enhance bioproduction and robustness to perturbations and antagonistic behaviors can be used to regulate population dynamics 42, 47, 74, 93.

Kong and colleagues recently reported a modular system for the rational engineering of any desired social interaction in synthetic *Lactococcus lactis* consortia by rearranging genetic components in a library of parts [29]. To generate competitive or parasitic social interactions, production of lactococcin A, a bacteriocin, was produced by a ‘predator’ strain and used to kill the ‘prey’. In cooperative behaviors, resistance mechanisms were incorporated, in which one or both organisms produce compounds that enable mutual survival. *E. coli* predator–prey systems have similarly been engineered by linking QS signals between strains to the production of a toxin or antidote [93] or, for cooperative behaviors, by inoculating two strains together in media with antibiotics, and linking QS signals produced by each member to the expression of an antibiotic resistance gene in the other [94].

As our understanding of the dynamic interactions within microbial ecosystems improves, so too will our ability to engineer microbial consortia with robust, stable, and predictable behaviors.

Alt-text: Box 1

Inducer molecules, such as Isopropyl β-D-1-thiogalactopyranoside (IPTG) or anhydrotetracycline (aTc), can be used to exogenously regulate transgene expression in organisms that express a corresponding, responsive promoter. Since genetic circuits and pathways distributed between members of a consortium are difficult to independently control due to a lack of gene regulatory systems without interference, orthogonal gene regulatory systems with minimal crosstalk have been developed 27, 28. To minimize crosstalk between an inducer and its regulated promoter, a transcriptional regulator can be engineered, or the IPTG-inducible promoter sequence to which it binds mutated.

Microorganisms can also be controlled exogenously with antibiotics and bacteriocins, which are peptides produced by some bacteria that inhibit the growth of closely related bacterial strains 26, 29. Each of these molecules can be genetically encoded or exogenously added to a growth medium, meaning they can be used to regulate cell populations within a microbial consortium in a cell–cell or environment–cell manner. At the same time, the required machinery to resist these molecules can be genetically encoded and its expression controlled by inducers or signaling molecules, which can be used to generate positive or negative interactions and produce desired social interactions between consortium members (Figure 1B) [29].

In addition to antibiotics, toxins, and inducer molecules, population-specific gene expression can be altered by tuning nutrient concentrations and culture conditions [30].

### Engineered Syntrophies to Build Codependent Strains

Metabolic interdependencies and cross-feeding is ubiquitous in natural microbial communities 31, 32, 33. Similarly, codependent synthetic consortia can be engineered via syntrophic interactions, in which organisms feed off of metabolites produced by other consortium members (Figure 1C) [34]. Typically, these mutually dependent consortia consist of co-auxotrophic strains whose survival is each dependent upon supplementation of the missing metabolite by another consortium member.

While co-auxotrophies are a viable approach for generating large communities of unique strains in a consortium, not all **auxotrophs** exhibit comparable growth patterns [35]. Specifically, a 14-member *E. coli* polyculture containing strains auxotrophic for different amino acids suggests that arginine, lysine, methionine, and threonine auxotrophs dominate a consortium after only a few days [35]. Organisms auxotrophic for nucleotide biosynthesis genes can also be used to construct codependent communities of *E. coli* [36].

Engineering eukaryotic synthetic consortia with co-auxotrophies is more challenging. Two nonmating strains of *S. cerevisiae* with lysine and adenine auxotrophies grow when cocultured, but demand that each strain is engineered to overproduce the metabolite required by the other [8]. To overcome the challenges associated with co-auxotrophic interactions between yeast, a self-established, metabolically cooperating yeast community (SeMeCo) was developed, in which metabolic auxotrophies were randomly introduced into a yeast population by loss of plasmids expressing genes involved in amino acid biosynthesis. These randomly introduced auxotrophies can be used to develop yeast communities that enter a state of syntrophic metabolic cooperation [37]. Co-auxotrophic interactions have also been engineered between a respiration-deficient yeast (via deletion of *cox2*, a mitochondrial gene encoding a subunit of cytochrome c oxidase) and endosymbiotic *E. coli* [38]. Here, the yeast provides thiamin to an *E. coli* auxotrophic for this vitamin and the *E. coli* shares ATP with the yeast host.

Computational models are also useful tools for designing syntrophic consortia (Box 2). Commonly based on economic frameworks, such as the biotic general equilibrium theory (BGET), these models predict metabolic networks based on the resource growth requirements, metabolic capabilities, and metabolite exchange rates between each consortium member [39]. The BGET framework is also useful for predicting growth rates of consortium members relative to the abundance of a given resource.

Box 2 **Computational Models for Designing and Predicting Behaviors of Microbial Consortia**

Engineering biological systems often requires accurate computational models, which enable faster iterations through the design–build–test–learn cycle [95]. Though most models in microbiology have focused on individual organisms, there is a growing body of literature specific to computational methods for designing microbial consortia, which often incorporate similar mathematical principles.

Dynamic models are one way to predict the behaviors of microbial consortia. These models can track a set of variables within a consortium over time, provided that the initial values and rates of change are known. They can be used to predict, for example, how the population of each member in the consortia will change in response to a given parameter, as in a predator–prey consortium 93, 96. This approach has also been used to predict temporal fluctuations in the mouse microbiota in response to antibiotic perturbations [97].

Stoichiometric models are also used to study or design microbial consortia and include methodologies that were initially developed for single cells. Flux balance analysis (FBA) is a method to simulate metabolic networks using genome-scale reconstructions [98]. Provided that the stoichiometric coefficient of each reaction is known, nearly any metabolic interaction within a microbial consortia can be modeled and adapted to specific environmental variations or resource availability [99]. FBA can be used to predict the growth rate of an organism when supplied with different energy sources or predict the rate at which a certain metabolite is produced. Metabolic networks of members in a consortia can also be studied in a variety of real-world contexts with elementary mode analysis, which breaks down each metabolic system into a series of biochemical pathways that satisfy steady-state conditions [100].

Finally, agent-based models offer a flexible computational approach to simulate the behaviors of microbial consortia over time. These models are particularly useful to model single cells within growing microcolonies. Modern agent-based simulators, including BSim2.0 and gro, can integrate the intracellular dynamics of each cell in the form of ordinary or delay differential equations, as well as predict cellular morphologies and physical interactions between each member in a consortium [101]. Some agent-based models can even be adjusted to account for complex conditions, such as environmental signals, nutrient uptake, and spatial separation between cells [102].

Alt-text: Box 2

## Synthetic Biology Tools Enable the Construction of Microbial Consortia with Defined Behaviors

**Figure 2:**
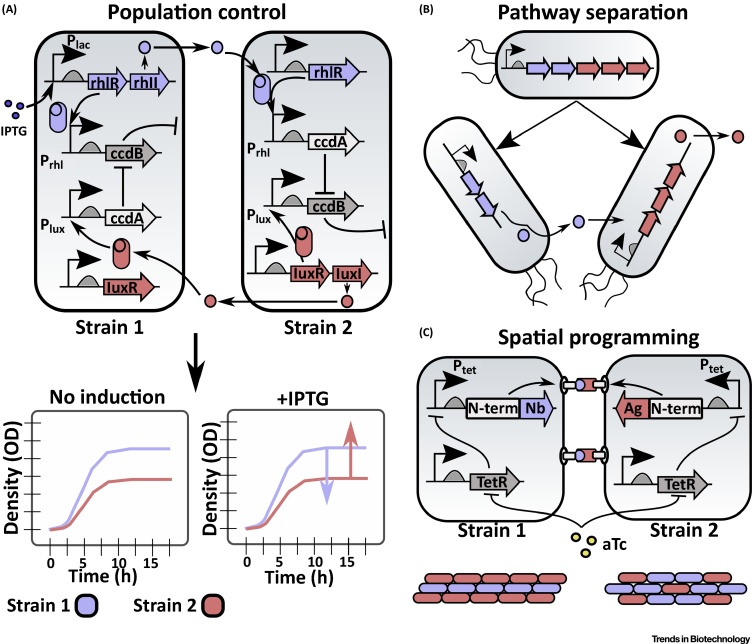
Engineering Behaviors of Synthetic Microbial Consortia. (A) The population of consortium members can be regulated by genetic circuits and feedback control. By linking quorum sensing (QS) systems (*rhl* and *lux*) to the repression of a toxin (ccdB) by an antitoxin (ccdA), strains in a consortium can be made to maintain a stable population ratio. In this example, addition of Isopropyl β-D-1-thiogalactopyranoside (IPTG) activates transcription of the P_lac_ promoter in strain 1, which increases the expression of ccdA and decreases the expression of ccdB in strain 2. This enables IPTG-inducible tuning of population ratios between the strains. Adapted from McCardell and colleagues [45]. Strain 2 similarly regulates the growth of strain 1. Unannotated promoters are constitutively expressed. (B) Heterologous pathways can be divided between consortium members. A five-gene heterologous pathway can be divided between two strains, the first of which expresses two of the heterologous genes to produce an intermediate metabolite. This intermediate may either diffuse or be transported to the other strain, which converts the intermediate to a desired end-product. (C) Spatial programming of at least two *E. coli* strains in a synthetic consortium can be achieved by engineering each to express either a nanobody (Nb) or corresponding antigen (Ag). An N-terminus (N-term) fusion mediates expression of the nanobody or antigen outside of the cell for cell–cell adhesion. TetR is constitutively expressed by both strains, which inhibits expression of the adhesion constructs until repressed by anhydrotetracycline (aTc). Expression of different Nb or Ag proteins can be used to form desired patterns between consortium members, such as layered or spheroid shapes (bottom). Adapted from Glass and Riedel-Kruse [54].

An understanding of the synthetic biology tools used to modify living organisms can also be applied to the engineering of yet more complex functions and behaviors in microbial consortia. Intercellular signaling, exogenous inputs, and syntrophic interactions are all tools that, together, can be applied to tune population levels, distribute or compartmentalize tasks, and define spatial morphologies in synthetic consortia (Figure 2).

### Controlling Population Levels

Intercellular signaling mechanisms, including QS, are useful for constructing bidirectional communication systems that, when interfaced with genetic circuits, can reliably control population levels within a mixed microbial consortium.

Methods to control population levels within a microbial consortium are useful in biotechnology applications where the relative production of a specific metabolite could affect the maximal titer of the end-product or cause toxicity to the chassis [40]. However, approaches to construct communities with user-defined population ratios demand stringently tested genetic circuits that produce replicable behaviors.

Synthetic circuits that regulate gene expression via orthogonal signaling molecules between two strains can be used to produce different population-level behaviors [41]. For example, cell populations in coculture can be engineered to express a different QS system that self-induces lysis (via expression of lysis gene E from bacteriophage X174) upon reaching a tunable population threshold [42], leading to oscillations in the population level of each strain.

In cases where consistent, rather than oscillatory, population ratios are desirable, feedback control can be used to continuously monitor and adjust a synthetic microbial consortium based on bidirectional communication between organisms [43]. By measuring the production of orthogonal QS molecules between organisms and then incorporating a feedback controller module that compares population levels, for example, the population of one member can be selectively reduced via a toxin/antitoxin system and population ratios between members in a consortium can be controlled [44].

Modeling results indicate that a two-strain consortium of *E. coli* can be engineered to maintain defined population ratios by linking orthogonal QS systems to the production of a toxin that increases in feedback strength as population discrepancies grow [45]. Each strain in this model produces a HSL molecule upon induction by IPTG or aTc. Each strain also expresses ccdB, a bacterial toxin, which is activated by its own HSL molecule. Inhibition of ccdB by ccdA, an antitoxin, is induced by the HSL molecule of the other strain (Figure 2A) [45]. By modifying the concentration of IPTG and tetracycline inducers, the population ratios between the two strains can be tuned.

### Pathway Separation and Distribution of Tasks

When an organism feeds off the resources produced by another member of the consortium, it may downregulate or abolish its own expression of the resource to minimize metabolic redundancies [46]. This syntrophic interaction (DOL) is also useful in synthetic microbial consortia because it can reduce the metabolic load, or burden, placed on any given organism 1, 4. DOL enables biosynthesis of metabolites normally too complex to produce from a single chassis, as entire heterologous pathways can be artificially segmented between organisms, in which each consortium member produces an intermediate compound that is used by the next organism in the supply chain (Figure 2B) [47]. Separating pathways between strains in a consortium is also effective for biomanufacturing applications, as organisms can be specifically selected for each stage of the pathway to enhance enzymatic activities and the relative production of each metabolite in the heterologous pathway tuned to avoid bottlenecks or toxic build-ups of intermediate metabolites 47, 48.

It is often difficult to select the genes from a heterologous pathway that should be expressed in each chassis for production of a desired end-product. Computational models provide a valuable tool to design synthetic consortia with optimal DOL schemes. A recent genome-scale metabolic model that incorporates reaction constraints, in which the number of intracellular and transport reactions in each strain are increasingly limited, suggests that some DOL strategies are also highly unintuitive, such as segmentation of the TCA cycle between two strains [49]. When confining each strain in a two-member *E. coli* consortium to only 11 transport reactions and 26 intracellular reactions (from core carbon metabolic pathways), for example, the model predicts that exchange of 2-oxoglutarate and pyruvate is a viable solution for growth of the coculture [49].

However, separating a biosynthetic pathway between organisms is not always optimal energetically, typically because the act of separating a pathway between multiple organisms may require additional enzymatic steps, such as heterologous transport proteins, if the intermediate metabolite does not freely diffuse or is not exported by passive transport mechanisms. In an *E. coli*–*S. cerevisiae* coculture, the production of plant benzylisoquinoline alkaloids requires seven heterologous enzymatic steps whereas, in *S. cerevisiae* monocultures, only four enzymes are required 50, 51. Computational models therefore suggest that DOL is beneficial for bioproduction from a synthetic consortium, relative to a monoculture, only if the biosynthetic pathway of interest reduces overall fitness in the case of high metabolic burden in the monoculture, if parts of the biosynthetic pathway take place outside of cellular confines, or if the end-product causes feedback repression of earlier enzymatic steps in the biosynthetic pathway [4].

### Programming Spatial Organization

Intercellular signaling mechanisms between cells and exogenous inputs to control gene expression can be used to form distinct spatial patterns and morphologies in synthetic microbial consortia by activating the expression of adhesins, ligand receptors, or other polymers.

In some natural consortia, organisms are found in biofilms or cell aggregates, which offer vast benefits for microbial ecosystems, including enhanced resistance to environmental perturbations and metabolic **commensalism** [52]. Brenner and Arnold studied this phenomenon with a two-member synthetic consortium and showed that spatial self-organization enhanced the growth advantage and resilience of biofilms to environmental disruptions relative to unstructured communities [9]. In biofilms, microbes produce adhesive cell surface proteins, called adhesins, together with extracellular polymers [53].

Genetically encoded cell–cell adhesion molecules can be used to program three-dimensional multicellular morphologies in synthetic consortia [54]. These synthetic adhesion molecules consist of a transcriptional regulator, an outer cellular membrane ‘tether’, and different pairs of adhesin and nanobody molecules that form specific interactions with one another. By coculturing an adhesin-presenting cell with a corresponding nanobody-producing strain, orthogonal, highly specific cell–cell adhesion was achieved and used to form different multicellular aggregates, including meshlike patterns with alternating cell-types, fibrous structures, and spheroid morphologies (Figure 2C) [54]. Expression of adhesin proteins from light-controlled promoters has also been used for lithography in *E. coli* biofilms at a spatial resolution of 25 μm [55].

Cells within biofilms are embedded in an extracellular polymeric substance (EPS) comprised of proteins, lipids, polysaccharides, and DNA, the latter of which can be actively secreted from living cells or released by cell lysis 56, 57. Since DNA sequences are highly amenable to engineering, they may offer a programmable method to define cell–cell spatial positions in a microbial consortium. Single-stranded DNA polymers with complementary sequences have been used to build self-assembling nanostructures functionalized with proteins [58]. Attachment of a DNA:human fibronectin protein nanostructure to HeLa cells, for example, enabled tunable control of cell morphology, signal transduction, and localization of transcription factors [58]. Designing functionalized DNA nanostructures for association and recognition between cells *ex vivo* may prove useful in the engineering of complex spatial morphologies within synthetic microbial consortia. The engineering of glycopolymers, which comprise a large portion of the EPS, could offer an additional approach to control cell–cell adhesion, since some bacterial adhesion processes are mediated by binding of cellular lectins (carbohydrate-binding proteins) to complementary carbohydrates [59].

## Selected Biotechnology Applications of Synthetic Microbial Consortia

The ability to control organisms within a consortium, divide labor, and manipulate spatial morphologies have been leveraged in numerous biotechnological applications. Synthetic consortia constructed with these methods have been applied for bioproduction, complex substrate utilization, and the assembly of functional **biomaterials**. However, the applications of synthetic microbial consortia discussed here are by no means exhaustive (Table 1).

**Table 1:** Tools and Approaches Used to Engineer Synthetic Microbial Consortia for Select Biotechnology Applications

Biotechnology application	Description	Typical organisms used	Synthetic biology tools	Synthetic biology approaches	Additional information	Refs
Degradation of complex substrates and pollutants	Defined interactions between consortia members for enhanced degradation of complex polymers, pollutants, or broad ranges of substrates	*E. coli* or *S. cerevisiae* cocultured with an organism possessing activity against pollutant or substrate	Computational models, engineered syntrophies, exogenous controllers	DOL, pathway separation	Examples commonly utilize a strain with natural enzymatic activity against target substrate (e.g., cellulose, pollutant) in coculture with a well-characterized chassis	63, 71, 73, 103, 104, 105, 106
Bioproduction of medicines, biofuels, and protein complexes	Synthetic microbial consortia as applied for bioproduction processes	*E. coli*, *S. cerevisiae*	Computational models, engineered syntrophies, exogenous controllers	Population control, DOL, pathway separation	Most common application of synthetic microbial consortia. Engineered signaling, DOL and pathway separation are typical approaches.	47, 50, 63, 67, 68, 103, 106, 107, 108
Functionalized biomaterials	Bidirectional communication in synthetic consortia for production of user-defined, functionalized biomaterials	*E. coli*, *B. subtilis*	Exogenous controllers, intercellular signaling	Spatial organization, DOL	Similar approaches to bioproduction, but demanding a higher level of spatial organization and bidirectional communication for producing biomaterials with defined patterns	76, 77, 106, 109
Distributed logic computing/ memory	Interfacing bidirectional communication with logic gates for consortia-wide computing or memory	*E. coli*	Intercellular signaling, computational models, exogenous controllers	Spatial organization, DOL	Logic gates distributed between consortium members enable more complex circuits with minimized burden placed on a single chassis. Logic gates are typically connected with QS or other signaling molecules.	83, 84, 110, 111, 112, 113
Biosensing	Detection of small molecules and metabolites with responsive, synthetic consortia	*E. coli, B. subtilis*	Intercellular signaling, exogenous controllers	DOL	The robustness and stability of microbial consortia may enable enhanced environmental biosensors with distributed logic and memory	114, 115

### Enhanced Bioproduction

The expression of large heterologous pathways in monocultures for bioproduction is challenging because the channeling of metabolic flux toward a desired end-product is often limited by metabolic burden, negative pathway effects (including toxicity of intermediates), metabolic crosstalk, and competition for intracellular resources [4].

Synthetic microbial consortia are particularly well-suited for the bioproduction of toxic or complex molecules because syntrophic interactions and DOL enables distribution of metabolic burden and modular engineering of heterologous pathways 47, 60. Distributing a heterologous pathway between organisms also enables each part of the pathway to be performed in an optimal cellular chassis [61]. Additionally, engineered microbial consortia typically exhibit enhanced biomass production relative to monocultures 62, 63.

*E. coli* and *S. cerevisiae* with compartmentalized tasks were used to produce the antitumor drug paclitaxel by expressing cytochrome taxadiene hydroxylase and a reductase in *S. cerevisiae*, while engineering the *E. coli* to secrete taxadiene, an isoprenoid. Cytochrome taxadiene hydroxylase is poorly expressed or has low activity in *E. coli*, and yeast commonly cannot achieve high production of isoprenoids alone 64, 65. Separation of this complex heterologous pathway between the organisms enabled an array of oxygenated taxadienes to be produced at nearly twice the titers previously reported 47, 66. Division of a heterologous pathway was also applied to *E. coli* polycultures expressing 15 different exogenous enzymes to produce anthocyanins from sugar for the first time outside of a native system [67].

While beneficial for producing a specified end-product and derivates thereof, separation of a heterologous pathway between strains is not particularly useful for producing end-products with many individual components. Toward this goal, each strain in a consortium could instead be engineered to function as a ‘specialized’ producer for one part of a full complex. The 34 proteins involved in prokaryotic translation were produced from polycultures of *E. coli* with this approach, by engineering each strain to function as a specialized producer of one protein component [68]. This required specific ratios between each population, however, to obtain the stoichiometries demanded for assembly of the translation machinery. Intercellular signaling and feedback circuits could potentially be used to enhance titers of functional, assembled complexes in future iterations.

### Expanded Substrate Usage

Though bioproduction from pure starting reagents has been well-demonstrated in metabolic engineering, tools to convert waste or inexpensive substrates into fine chemicals without energy-intensive purification processes are desirable [69].

Oftentimes, the use of cheap carbon sources requires complex metabolic pathways comprised of a repertoire of enzymes and cofactors, some of which are uncharacterized or unknown [70]. This limits their transferability and expression in a desired chassis. In addition, complex substrates usually comprise a milieu of carbon sources (e.g., hexoses, pentoses, alcohols, and acids), which makes it difficult or impossible to adapt a single bioproduction strain to utilize all of them at the efficiencies required for efficient bioprocessing [69]. Modularity of cells within a microbial consortium and the compartmentalization of tasks enables unprecedented ranges of substrate usage in synthetic microbial consortia relative to monocultures.

Due to the modularity of synthetic microbial consortia, individual populations can be engineered to function as ‘specialized’ strains that perform only one function toward a greater goal. This is particularly useful for fermenting multiple carbon sources simultaneously. Consortia of specialized *E. coli* strains have been engineered to each consume a single carbon source by deleting genes encoding transporters and enzymes involved in the utilization of other carbon sources [71]. This approach of specialized populations enabled a mixture of arabinose, glucose, xylose, and acetate, prevalent substrates in lignocellulose, to be fermented at rates significantly greater than ‘generalist’ strains [71]. The conversion of lignocellulosic sugars into high value products is additionally hindered by the competitive inhibition of D-xylose transporters by D-glucose [72]. A three-strain consortium of *S. cerevisiae* that ferments glucose, xylose, and arabinose overcame this limitation by engineering each strain to ferment only one of the three sugars, resulting in a consortium significantly more stable than a generalist strain [73].

*Synechococcus elongatus*, a photosynthetic cyanobacterium, has also been engineered to export up to 85% of its photosynthetically fixed carbon in the form of sucrose by heterologous expression of *cscB*, a proton/sucrose symporter. The sucrose secreted by this strain (*cscB+*) is enough to sustain the growth of *B. subtilis*, *E. coli*, and *S. cerevisiae* [74]. Though there is no direct benefit for *S. elongatus* in this consortium, coculturing of *cscB+* with an *E. coli* strain expressing genes involved in poly-beta-hydroxybutyrate (PHB) biosynthesis enabled the production of PHB from photosynthetically derived sucrose.

### Production of Functional Biomaterials

The compartmentalization of tasks, modular tenability, and intercellular signaling mechanisms between members of synthetic microbial consortia make them an ideal platform to produce complex, functional biomaterials. Biomaterial production necessitates fine-tuned control of gene expression and pattern formation. Advancements in synthetic biology tools for spatial programming will enable synthetic consortia to produce materials of defined sizes, shapes, and patterns.

Curli fibrils are an extracellular amyloid produced by many strains of Enterobacteriaceae, including *E. coli*. Curli production is encoded by two divergently transcribed operons, *csgBA* and *csgDEFG* [75]. An *E. coli* consortium has been engineered to produce curli fibrils of defined patterns and properties by controlling expression of the *csg* operons with QS and inducible gene expression systems [76]. By mixing HSL ‘sender’ cells that produce curli under the control of a tetracycline-responsive promoter with HSL ‘receiver’ cells that produce curli in response to signals from the sender, autonomous patterns of amyloid emerge [76]. Intriguingly, these curli fiber patterns change over time and their properties can be tuned as a function of the population ratio between the cell-types and induction with aTc. Patterning of curli fibers at the nanoscale was achieved by linking curli protein subunits together in tandem to form fibrils with syncopated, repeating patterns of a defined length. These fibers can also be interfaced with diverse inorganic materials to create functionalized materials, including conductive curli biofilms that sense and respond to environmental signals and nanowires with embedded gold particles [76].

Extracellular protein conjugates are another type of biomaterial enhanced by the modularity inherent within synthetic microbial consortia. *Ex vivo* protein conjugates with functional properties can be produced by engineering each consortium member to secrete one protein fused to a peptide tag, which form covalent bonds upon interaction with other tagged proteins. The compartmentalized production of ‘interlocking’ proteins has been demonstrated in both *B. subtilis* and *S. cerevisiae* strains to form functional biomaterials with activity against xylan and cellulose 77, 78.

In the future, intercellular signaling between members of a consortium, together with the ability to program spatial patterns via cell–cell adhesion molecules, will enable the production of biomaterials with highly complex shapes, patterns, and textures, akin to those found in natural environments.

## Concluding Remarks and the Future of Synthetic Microbial Consortia

Though synthetic microbial consortia already enable improvements for some biotechnological applications, there are still substantial research questions to be addressed (see Outstanding Questions). Future advancements in the engineering of microbial consortia will mirror the development of improved synthetic biology tools, including capabilities in genome engineering and orthogonal communication systems, and the study of natural microbial ecosystems with high-throughput methods, such as metaproteomics and metametabolomics, as well as single-cell analysis [79]. By uncovering the principles governing natural microbial ecosystems, we will be better equipped to develop new tools and methodologies for their rational engineering.

‘Nonmodel’ organisms dominate natural communities of microorganisms and typically possess unique enzymatic or fitness advantages that may further enhance both the properties of synthetic microbial consortia (e.g., increased robustness) and the efficiency of bioprocesses [80]. However, many nonmodel organisms are difficult to culture in laboratory settings or intractable to genetic engineering, making it difficult to study and access their biosynthetic potential [81]. In part, this challenge is being addressed with emerging CRISPR/Cas tools, enhanced computational models, and complementary advancements in synthetic biology methods, including DNA assembly, automation, and computer-aided design, which is enabling the rapid construction, testing, and characterization of genetic parts from diverse organisms [82].

**Figure 3:**
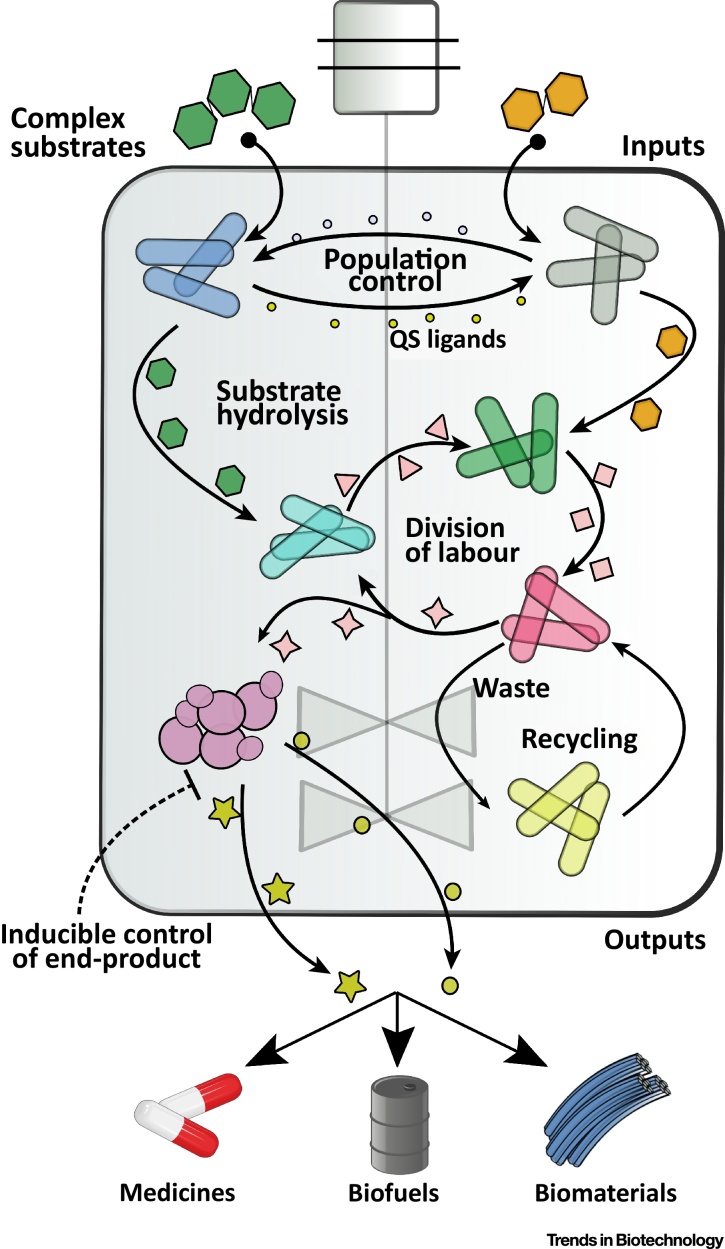
Key Figure: The Potential of Synthetic Microbial Consortia in Bioprocesses of the Future

The development of orthogonal, interdomain communication channels and an improved understanding of metabolic cross-feeding networks that remain stable over time will also enhance our ability to compartmentalize complex tasks and functionalities in consortia engineered for bioproduction (Figure 3, Key Figure), collective memory (in which logic gates engineered into synthetic consortia are used to record external inputs) 83, 84, or for dynamic therapeutic applications in the human gut microbiome [85]. While individual species have been engineered for therapeutic applications *in vivo* 86, 87, it is only recently that synthetic microbial consortia, induced by nutrient and dietary perturbations, have been engineered to coexist in the mouse gut [88]. We anticipate that in the next number of years, more works will be published on the engineering of human, animal, or plant microbiomes for improved immunity and nutrition. The future of engineered microbial communities is fast approaching and will represent a new architectural level of synthetic biology and metabolic engineering, where *ad hoc* communities are made by design.

Outstanding Questions How can we best learn from natural communities to facilitate the design and engineering of *ad hoc* synthetic consortia with desirable features? How do natural communities maintain robustness and stability within changing environments?

How many members of a community can be engineered while maintaining predictability and control? Is there a theoretical limit to the complexity of synthetic microbial consortia? Which approach is best suited for constructing a consortium with many members?

How can advancements in feedback control for defined population ratios between consortium members best be used to enhance bioproduction processes?

Will new computational tools enable the modeling and rational design of microbial consortia based on a knowledge of exchanged metabolites? How will advancements in computing power facilitate the modeling of increasingly complex microbial communities?

How will emerging methods in adhesin engineering or other polymer engineering approaches enable more precise spatial morphologies to build biomaterials of increasing complexity?

How can we best apply synthetic microbial consortia to study natural communities and organisms that cannot survive in monocultures?

How does crosstalk between intercellular signaling mechanisms, such as quorum sensing, impact community dynamics and robustness?

Will we be able to design and implement general principles to establish interspecies communities, or will the tools used to engineer consortia vary between species and domains?

Will modern analytical tools, including single-cell studies, enhance our understanding of communication and metabolite exchange within microbial consortia? Can we use this information to develop intercellular signaling mechanisms between nonbacterial and/or nonmodel organisms?

## Glossary

**Amensalism**: a biological relationship in which one organism is impaired and the other is unaffected.
**Autoinducer**: a signaling molecule that is produced in response to a change in the density of a cell population. The concentration of an autoinducer increases as population density increases and regulates gene expression upon reaching a minimal threshold.
**Auxotrophy**: the inability of an organism to synthesize a compound or metabolite that is required for its growth. Auxotrophic organisms can grow if this missing metabolite or compound is supplemented in its growth media, or if another member in the consortia produces the compound and shares the resource.
**Biomaterial**: any material produced by a living organism. Cellulose, keratin, and chitin are common examples.
**Burden**: the molecular load placed on an organism, typically caused by the introduction of exogenous genes. Burden often reduces the growth rate of an organism.
**Chassis**: the host cell that is being genetically modified. This term is commonly used in synthetic biology and is canonically used to refer to model organisms for which abundant characterized genetic parts are available for its engineering.
**Commensalism**: a biological relationship in which one organism benefits and the other is unaffected.
**Microbial consortia**: a grouping of microorganisms, either from different species or the same species but different strains, in which members interact with one another by sharing nutrients, cross-feeding, or by performing engineered behaviors. In this review, we use this term interchangeably with microbial community.
**Modular**: a functional unit, such as a transcriptional unit, that maintains its properties and function regardless of its context or what it is connected to.
**Monoculture**: a microbial culture that consists of a single type of organism.
**Natural consortia**: any community of organisms isolated from a native ecosystem, but otherwise unmodified from their native context.
**Neutralism**: a biological relationship in which organisms interact, but neither help nor harm one another.
**Orthogonal**: a functional unit or interaction whose structure or function is dissimilar to those found in nature, and so does not interact with the native unit. Orthogonality between quorum sensing systems, for example, is important in synthetic biology to ensure that promoters are not regulated by multiple signals.
**Synthetic microbial communities**: a community of organisms composed of two or more genetically engineered cell populations. The word ‘community’ in this review is interchangeable with consortium.
